# A study of “left against medical advice” emergency department patients: an optimized explainable artificial intelligence framework

**DOI:** 10.1007/s10729-024-09684-5

**Published:** 2024-08-13

**Authors:** Abdulaziz Ahmed, Khalid Y. Aram, Salih Tutun, Dursun Delen

**Affiliations:** 1 Department of Health Services Administration, School of Health Professions, The University of Alabama at Birmingham, Birmingham, AL 35233 USA; 2 Department of Biomedical Informatics and Data Science, Heersink School of Medicine, University of Alabama at Birmingham, Birmingham, AL 35233 USA; 3 School of Business & Technology , Emporia State University, Emporia, KS 66801 USA; 4 WashU Olin Business School, Washington University in St. Louis, St. Louis, MO 63130 USA; 5 Center for Health Systems Innovation, Department of Management Science and Information Systems, Spears School of Business, Oklahoma State University, Stillwater, OK 74078 USA; 6 Department of Industrial Engineering, Faculty of Engineering and Natural Sciences, Istinye University, Sariyer/İstanbul,, 34396 Türkiye

**Keywords:** Left against medical advice (LAMA), Predictive analytics, Machine learning, Simulated annealing, Emergency department, Explainable AI

## Abstract

The issue of left against medical advice (LAMA) patients is common in today’s emergency departments (EDs). This issue represents a medico-legal risk and may result in potential readmission, mortality, or revenue loss. Thus, understanding the factors that cause patients to “leave against medical advice” is vital to mitigate and potentially eliminate these adverse outcomes. This paper proposes a framework for studying the factors that affect LAMA in EDs. The framework integrates machine learning, metaheuristic optimization, and model interpretation techniques. Metaheuristic optimization is used for hyperparameter optimization-one of the main challenges of machine learning model development. Adaptive tabu simulated annealing (ATSA) metaheuristic algorithm is utilized for optimizing the parameters of extreme gradient boosting (XGB). The optimized XGB models are used to predict the LAMA outcomes for patients under treatment in ED. The designed algorithms are trained and tested using four data groups which are created using feature selection. The model with the best predictive performance is then interpreted using the SHaply Additive exPlanations (SHAP) method. The results show that best model has an area under the curve (AUC) and sensitivity of 76% and 82%, respectively. The best model was explained using SHAP method.

## Highlights

- Adaptive Tabu simulated annealing is utilized to optimize machine learning.
- The hyperparameters of Xgboost are optimized.
- Factors affect LBTC patients are studied.
- Extensive experiments are conducted to show the robustness of the proposed models.

## Introduction

Emergency departments (EDs) are the main source of hospital admissions [1]. When a patient visits an ED, different disposition modes are possible, including admission as an inpatient, discharged, expired, or transferred. Another important disposition status is when the patient leaves before receiving medical care, during treatment, or against medical advice. Those patients are called left without being seen (LWBS), left subsequent to being seen (LSBS), and Left against medical advice (LAMA), respectively [2–5]. The Fourth Emergency Department Benchmarking Alliance (EDBA) summit published a metric called left before treatment complete (LBTC), to refer to LWBS, LAMA, and LSBS combined [5].

Leaving the ED before finishing treatment is associated with a higher risk of readmission and mortality [6, 7]. Those patients are often severely ill and when they leave without receiving appropriate or timely medical care, their life might be at risk. Leaving prior to receiving formal discharge recommendation from a physician, AKA LAMA, is not an uncommon situation in EDs. LAMA is observed in approximately 1.3%-2.8% of all hospital discharges. Patients who leave against medical advice have been shown to have an elevated incidence of subsequent readmissions and medical complications. Literature indicates that such patients consume additional healthcare resources, resulting in escalated financial burdens on healthcare systems compared to those who complete standard discharge protocols [8]. Therefore, investigating the factors that affect LAMA rate is crucial to improving the quality of care, efficiency, throughput, and revenue.

The purpose of this study is to develop a machine learning model that predicts LAMA patients and discriminates them from patients who leave after being seen and treated (SAT). By leveraging both triage data and demographic information, this study develops a machine learning model to predict the likelihood of patients to leave against medical advice. This predictive capability is intended to provide hospitals with the insights required to formulate effective strategies, ultimately reducing the occurrence of LAMA instances, and enhancing patient care outcomes. In the past few years, machine learning methods have been used in many healthcare applications related to performance improvement in the areas of emergency care [9], organ transplant [10], and mental health [11]. One of the most challenging tasks in developing machine learning models is that most algorithms have many parameters that need to be tuned to achieve desirable performance [9]. In this paper, we propose a new approach that integrates adaptive tabu simulated annealing (ATSA) optimization algorithm with eXtreme Gradient Boosting (XGB). The new algorithm is called ATSA-XGB. The goal of ATSA is to optimize the parameters of XGB and then use the optimized model for predicting LAMA patients. This study contributes to the theory and practice of healthcare analytics, the application of which creates value for healthcare operations. This study also contributes to the area of integrating machine learning and optimization by demonstrating how machine learning performance can be boosted using optimization techniques. More specifically, the theoretical and practical contributions of this study are as follows:

- An explanatory machine learning approach is proposed to study the factors that affect the rate of LAMA patients.
- A new approach is proposed to optimize the parameters of XGB based on ATSA.
- To the best of our knowledge, this is the first study that proposes ATSA for optimizing the hyperparameters of machine learning. Also, this is the first study that develops a comprehensive machine learning framework to predict LAMA status.
- Extensive experiments were conducted to evaluate the proposed ATSA-XGB algorithm.

The remainder of this paper is organized as follows. Section 2 summarizes the relevant studies on LAMA. Section 3 describes the proposed methodologies including data collection, preprocessing, and model development. Section 4 presents experimental results. Finally, Section 5 summarizes the conclusions and outlines future work.

## Literature review

Several studies proposed models to predict the disposition status of ED patients. Studies have mainly focused on the most common disposition statuses, which are admission and discharge [9, 12–18]. Studies have also investigated the reasons and consequences of LAMA patients. Table 1 provides a summary of relevant LAMA, LWBS, and LBTC studies. Most of the studies focused on either LWBS or LBTC in general, except for one study that compared LWBS and LAMA patients. Most of the studies performed univariate analysis. In all the univariate studies, the comparisons were based on patient demographics, medical conditions, and hospital characteristics. For example, [20] conducted a univariate analysis to investigate whether LWBS patients needed immediate treatment after leaving the ED. They used patient demographics such as age, sex, race, and other information including insurance status, chief complaint, and acuity level to perform the comparison. [21] used univariate analysis to investigate if the LWBS patients receive alternative medical care after leaving. Although univariant analysis is simple and easy to understand, it is not comprehensive and does not consider the relationship between the different variables. Therefore, some studies used multivariate approaches to study LWBS, LBTC, and/or LAMA patients.

**Table 1:** Summary of the works that studied LWBS, and LBTC

Study	Sample size	Univariate Study	Multivariate study	Machine learning study	Predictors/characteristics	LWBS	LBTC	LAMA
Baker et al. [20]	186 + 211	*			8	*		
Sheraton et al. [25]	32,680,232	*	*	*	13	*		
Improta et al. [26]	83,739			*	5	*		
Mohsin et al. [21]	14 741	*			8	*		
Sun et al. [27]	810.6 M	*			8	*		
Carron et al. [19]	307,716	*			5	*		*
Ding et al. [28]	3,624	*			10	*		*
Mataloni et al. [6]	835,440	*			12	*	*	
Crilly et al. [29]	64292	*	*		8	*		
Tropea et al. [7]	239,305	*	*		9	*		
Goodacre and Webster [30]	71331		*		6	*		
Pham et al. [31]	283,907	*	*		6	*		
Hitti et al. [32]	266	*	*		8	*		
Zubieta et al. [33]	42,750	*			4	*		
Mohsin et al. [34]	4,356, 323	*	*		11	*		
Smalley et al. [5]	626,548	*			4		*	
Arab et al. [35]	768	*			9		*	
Natan et al. [36]	390	*	*		5		*	
Pages et al. [37]	2425	*	*		7			*
Mitchell et al. [22]	561,823	*	*		24			*
Adeyemi and Veri [38]	219564	*	*		10			*
Jerrard and Chasm [39]	199	*			4			*
Myers et al. [40]	581,380	*			8			*
Villarreal et al. [8]	546,856	*			11			*
Our study	478,212			*	17			*

Few studies conducted multivariate analysis, but most of them utilized logistic regression to study LBTC, LAMA, and/or LWBS patients. For instance, [22] used a logistic regression model to identify the factors that affect the rate of LAMA or LBTC patients. Twenty factors were considered including patient diagnosis, race, gender, education level, and vital signs. Although logistic regression can capture the relationship between multiple inputs and a binary output, its performance declines as the number of features increases [23]. In our study, we combine machine learning and optimization to develop a high-accuracy model to identify and explain the factors that affect the rate of LAMA patients. With machine learning, complex nonlinear relationships among the various variables can be captured [24]. Machine learning methods overcome the problem of noisy data and can handle different types of variables including continuous, discrete, and text data [10]. Few studies developed machine learning models to analyze the factors affect either LWBS or LBTC, but most of them are not explainable. Among the previous studies, no study utilized machine learning to study the factors that affect LAMA. Our study provides both a tool to predict LBTC patients and an interpretation of the features that affect LBTC disposition status.

## Methodology

This section presents the different parts of the research framework including data preprocessing, feature selection, model development, and model interpretation.

### Research framework

**Fig. 1:**
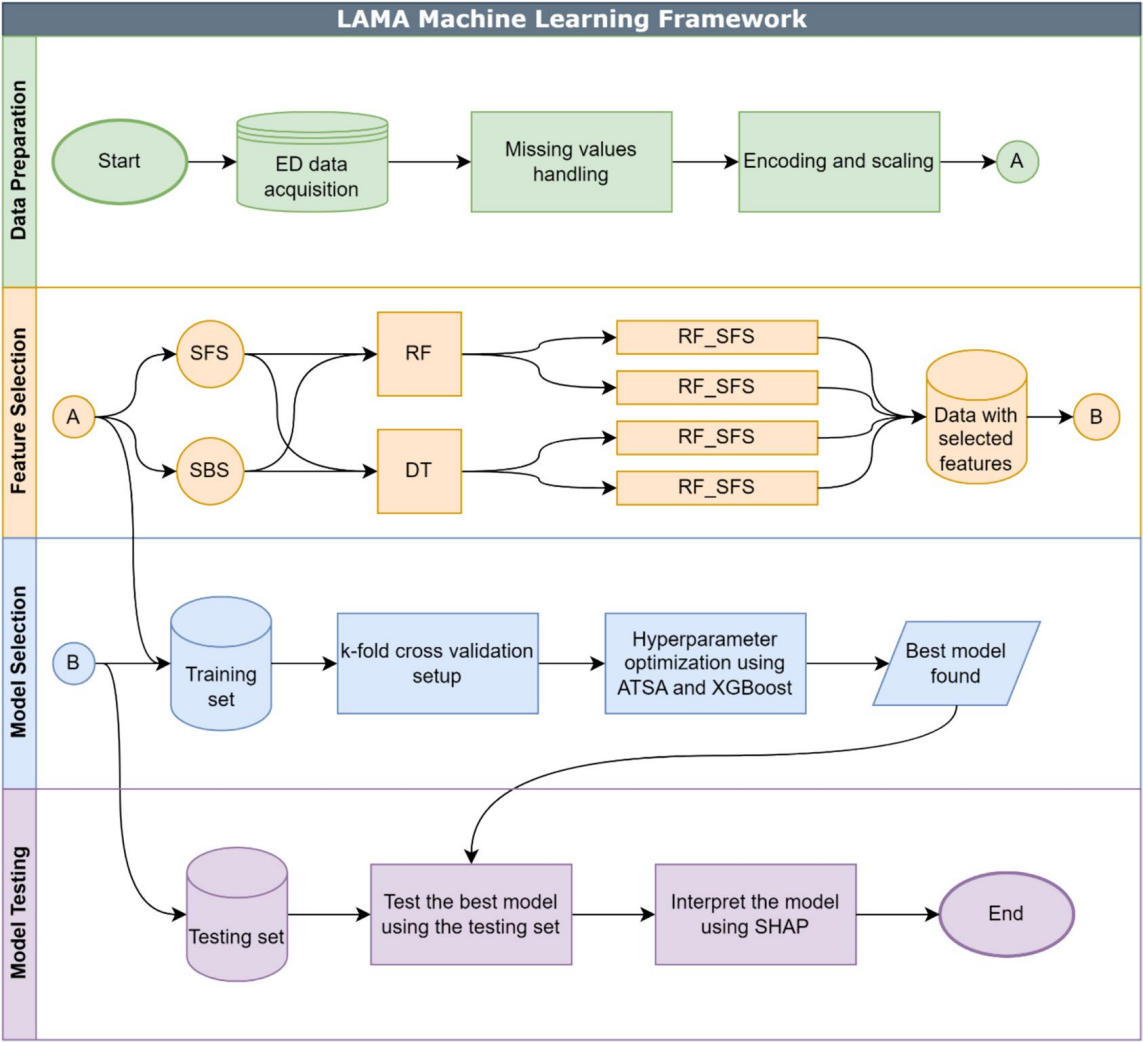
Proposed research framework

Figure 1 illustrates the proposed framework. First, the data is obtained and preprocessed. The data includes all the events that describe an ED episode from arrival to discharge. In the preprocessing stage, the missing values are handled, and the features are encoded and scaled. In the feature selection stage, two machine learning models are used: Random Forest (RF) and Decision Tree (DT). These two models are wrapped with two feature search algorithms: Sequential Forward Selection (SFS) and Sequential Backward Selection (SBS). Five data groups result from the feature selection step. Four groups are based on the feature selection methods, and one includes all the features, (i.e., X_all). Each data group is used to train and test predictive models during the model development. Each data group is split into two sets: training and testing. An 80% to 20% training-to-testing ratio is used. The models are then trained using k-fold cross validation. The best model is then tested using the testing set. Due to data imbalance, random oversampling and undersampling are utilized during the training phase. The test set is kept as is. ATSA is used to optimize the parameters of XGB during model training with k-fold cross validation. Finally, the model with the highest testing performance is interpreted using SHAP.

### Data collection

Retrospective patient-level data of ED visits between 2017 and 2019 was collected from a partner hospital located in the Midwest of the United States. The data includes more than 450 k ED visits and more than 32 predictors. The data was collected from four locations of the partner hospitals. The Sanford Health facilities demonstrate varying capacities across four locations. Location 1 has 84 ED beds, manages over 180,000 annual patient visits, and has 583 inpatient beds. Location 2 is equipped with 58 ED beds, accommodates over 120,000 annual patient visits, and offers 462 inpatient beds. Location 3 has 34 ED beds, handles over 50,000 patient visits each year, and provides 166 inpatient beds. Finally, Location 4 includes 26 ED beds, serves over 30,000 patients annually, and has 147 inpatient beds.

Since this study proposes a model that predicts LAMA patients at the time of triage, we only included a combination of triage data and demographic information. We have intentionally opted not to incorporate medical diagnoses as a feature into the proposed model, as the objective is to enable predictions before a patient sees a physician and following the initial assessment of vital signs. Also, this model is static, by which the prediction is performed once at the time of triage. This approach ensures that our predictions can help hospitals to take early measures to prevent LAMA incidents. The original data described in Table 2 consist of a total of 19 features, four of which are categorical, and the rest are numerical. ED Disposition in the table is the response variable which includes the two output classes, SAT and LAMA, which are predicted using the proposed machine learning framework. We used one-hot encoding to preprocess the categorical features. For instance, if a feature has $n$ categories, it is replaced by $n - 1$ binary features. The following inclusion and exclusion criteria are applied to prepare the data for modeling:

- The timestamp features are excluded such as arrival and physician assessment times.
- The arrival time feature is converted into three features: month (e.g., January) and hours (e.g., 1–24). The minutes and the seconds are excluded.
- The demographic and vital signs features are included. For vital signs, if a patient has multiple vital signs, the average is taken.
- Waiting time feature is calculated as the time between patient arrival and the time when a patient sees a nurse.

**Table 2:** Summary of original ED data

Feature	Average ± standard deviation (range) for numerical features, % for categorical features
ED Location ID	
10001	27.61%
10025	16.22%
10026	20.32%
15001	35.86%
Age	42.46 ± 24.83 (0–107)
Sex	
Male	53.52%
Female	46.48%
Zip Code	(10016, 99750)
Ethnicity	
Not Hispanic or Latino	4.78%
Hispanic or Latino	95.22%
BMI	28.56 ± 7.804 (7.38–50.68)
Patient Smoking Status	
Current Smoker	23.63%
Former Smoker	1.07%
Never Smoker	5.29%
Unknown	70.00%
Systolic Blood Pressure	121.01 ± 15.016 (69–168)
Diastolic Blood Pressure	72.99 ± 10.802 (35–107)
Pulse Rate	82.71 ± 16.051 (34–130)
Temperature in Fahrenheit	97.87 ± 0.8 (94.4–100.6)
ED Disposition	
LAMA	0.66%
SAT	99.34%
O2 Saturation	97.54 ± 1.968 (92–100)
Respiratory Rate	16.92 ± 2.122 (12–23)
ESI	3.23 ± 0.633 (2–5)
Day of the Month	15.71 ± 8.778 (1–31)
Month of the Year	6.51 ± 3.433 (1–12)
Hour of the Day	13.7 ± 6.175 (0–23)
Waiting Time	9.31 ± 11.579 (0–51)

### Handling missing values

**Fig. 2:**
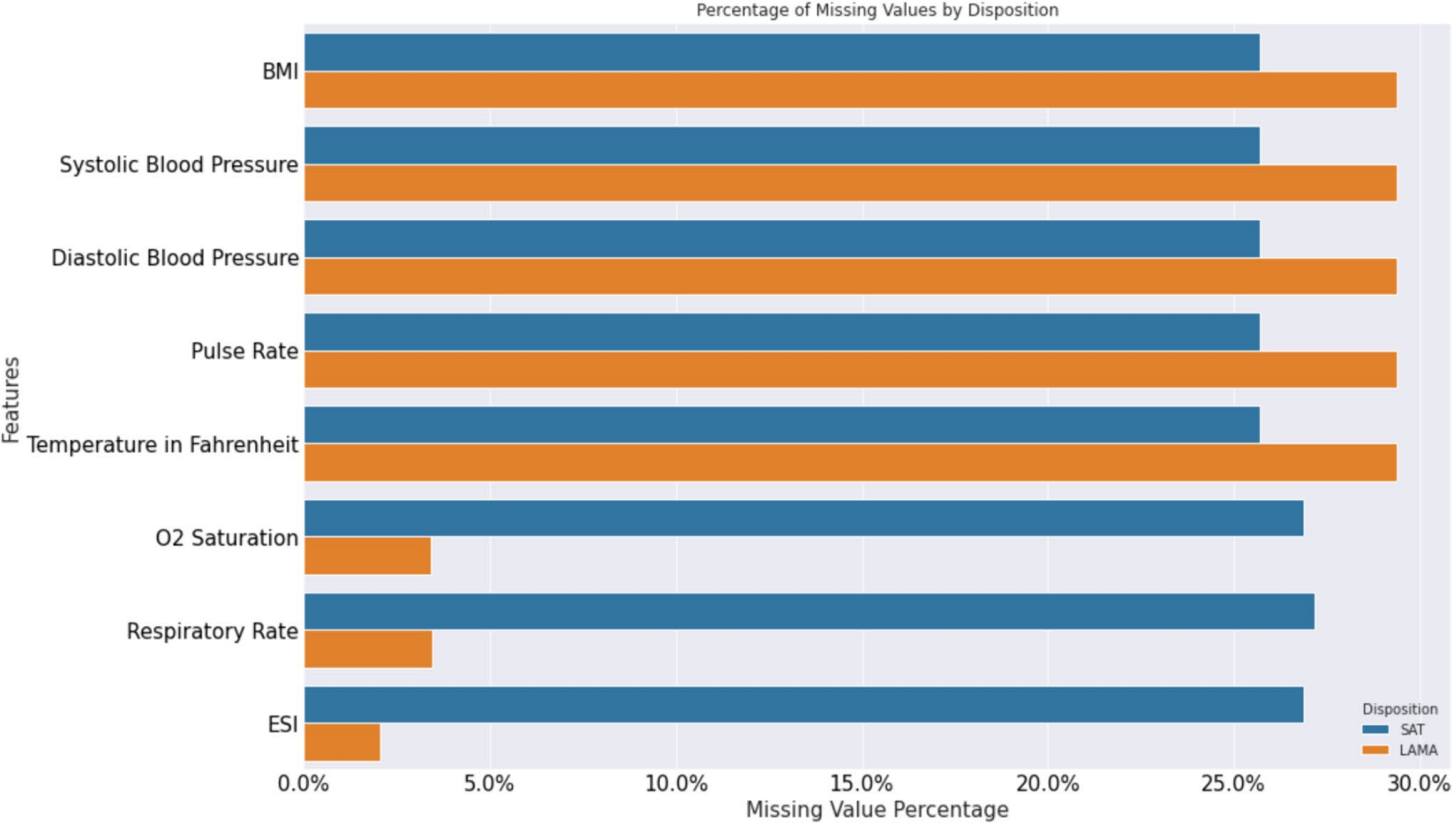
% of missing values per disposition class (SAT and LAMA)

Figure 2 shows the percentage of missing values per output classes (i.e., LAMA and SAT). For most features, the LAMA disposition exhibits a greater percentage of missing values compared to SAT, such as BMI and Pulse Rate. Conversely, for certain features such as Respiratory Rate and O2 Saturation, the SAT class demonstrates a higher percentage of missing values compared to the LAMA class.

After encoding categorial features, we conducted data imputation to handle missing values. If all the visits with missing values are deleted, a significant amount of information will be lost. Therefore, K-Nearest Neighbor (KNN) is used for imputation. With a KNN imputer, missing values are replaced by the average of $K$ neighbors. We employ a validation method to assess the reliability of the KNN imputation technique in handling missing data. We dropped all rows with missing values to obtain a subset of the data with no missing values. We then introduce artificial missingness by randomly replacing 10% of the values across all features in the complete data subset. We then apply KNN imputation to this dataset with artificially induced missing values. We then evaluate its performance by calculating the Mean Absolute Percentage Error (MAPE) between the original values in the complete data subset and the imputed values in the artificial subset, focusing specifically on the artificially introduced missing values across selected features of interest. This targeted evaluation enables us to derive an understanding of the imputation accuracy, ensuring a thorough and reliable validation of the KNN imputation method. The outcomes of this process are crucial, providing empirical support for confidence in our imputed dataset and significantly contributing to the robustness and reliability of this study. After data imputation, categorial features are encoded using one-hot encoding. The output feature (disposition decision) includes two categories: SAT and LAMA patients. Table 3 shows a description of the processed dataset which was used to train and test the proposed machine learning framework.

**Table 3:** Summary of processed ED data

Feature (category code)	Average ± standard deviation (range) for numerical features, % for categorical features
Age	42.46 ± 24.83 (0–107)
Zip Code	(10016, 99750)
BMI	28.34 ± 7.11 (7.38–50.68)
Systolic Blood Pressure	119.70 ± 13.83 (69.0–168)
Diastolic Blood Pressure	72.37 ± 9.77 (35.0–107)
Pulse Rate	83.95 ± 14.41 (34.0–130)
Temperature in Fahrenheit	97.85 ± 0.64 (94.4–100.6)
ED Disposition	
LAMA (1)	0.66%
SAT (0)	99.34%
O2 Saturation	97.39 ± 1.76 (92.0–100)
Respiratory Rate	17.02 ± 1.86 (12.0–23)
ESI	3.21 ± 0.56 (2–5)
Day of the Month	15.71 ± 8.778 (1–31)
Month of the Year	6.51 ± 3.433 (1–12)
Hour of the Day	13.7 ± 6.175 (0–23)
Waiting Time	9.31 ± 11.579 (0–51)
ED Location ID 10025	
10025 (1)	16.22%
Other (0)	83.78%
ED Location ID 10026	
10026 (1)	20.32%
Other (0)	79.68%
ED Location ID 15001	
15001 (1)	35.86%
Other (0)	64.14%
Sex	
Male (1)	46.48%
Female (0)	53.52%
Ethnicity	
Not Hispanic or Latino (1)	95.22%
Hispanic or Latino (0)	4.78%
Patient Former Smoker	
Former Smoker (1)	1.07%
Other (0)	98.93%
Patient Never Smoker	
Never Smoker (1)	5.29%
Other (0)	94.71%

### Handling data imbalance

Oversampling and undersampling are utilized to address this issue of imbalanced number of instances across the two output classes. Oversampling increases the size of underrepresented classes by duplicating existing instances or creating new synthetic ones to improve model performance on these classes. Undersampling, on the other hand, reduces the size of overrepresented classes by randomly removing instances to balance the dataset but potentially leading to information loss. These methods aim to prevent model bias towards the majority class. The selection between these two methods depends on the dataset's specific characteristics.

### Feature selection

Feature selection is an important step of model development. The goal of feature selection is to reduce the computational cost of model development and improve the generalization of models by excluding irrelevant features [41]. In this paper, sequential feature selection methods (SFMs) are used for feature selection. The SFMs are greedy search algorithms used to select a subset of $k$ features from a dataset composed of *d* features, where $k < d$. The features are added or removed sequentially based on model performance. Every time a feature is added or removed; the model performance is evaluated. The process continues until the model performance converges. Two sequential feature selection methods are used in this paper: 1) SFS; 2) SBS. The two methods are used along with two machine learning-based feature selection methods, which are DT and RF.

Consider a dataset with *d* features (${X= x}_{1}, { x}_{1}, \dots , {x}_{d}$) and ${\overline{X} }_{k}$ is the subset of selected features, where ${\overline{X} }_{k}=\left\{{\overline{x} }_{j} \right|j=1, 2, .., k;{\overline{x} }_{j} \in X \}$ and$K=\text{1,2},\dots d$. The pseudocode codes for SFS and SBS are shown in Algorithms 1 and 2, respectively. In SFS, a model starts with zero features such that${\overline{X} }_{0}= \varnothing , k=0$. In each iteration, a feature is added to the list of selected features ${\overline{X} }_{k+1}= {\overline{X} }_{k}+{\overline{x} }^{+}$ and then the model is evaluated. If the model performance is improved, the feature is added and otherwise, it is excluded. The process continues until a termination criterion is met. In SBS, the model starts with all features${\overline{X} }_{0}= X, k=d$. In each iteration, a feature is removed from the list of selected features $({\overline{X} }_{k-1}= {\overline{X} }_{k}-{x}^{-})$ and the model performance is evaluated. The process continues until a termination criterion is met.

**Figure:**
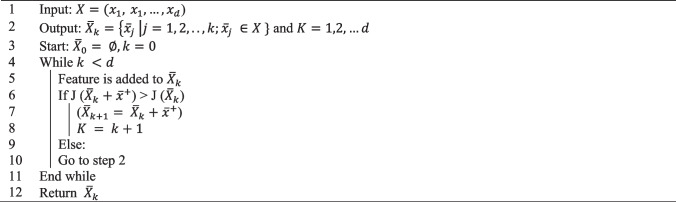
**Algorithm 1**- SFS feature selection

**Figure:**
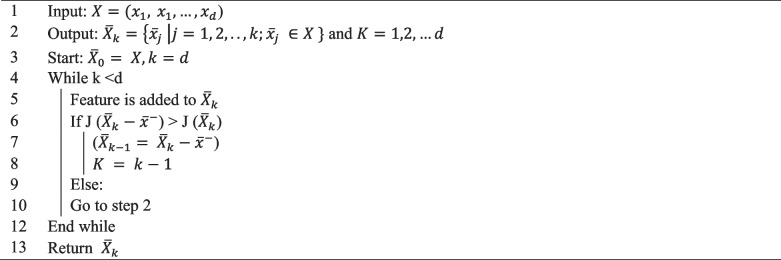
**Algorithm 2**- SBS feature selection

### Extreme gradient boosting (XGB)

XGB, developed by Chen and Guestrin [42], is a gradient boosting algorithm. It has competitive prediction performance and computational time compared to other well-known machine learning algorithms (Chen et al. 2020).

XGB obtains a strong learner (i.e., a tree), based on the sum of predictions of multiple weak learners. This process can be summarized using the following equation:

1 $${\widehat y}_i=\varphi\left(x_i\right)=\begin{array}{cc}\sum\limits_{k=1}^Kf_k(x_i),&f_k\in F\end{array}$$

where ${x}_{i}$ is a vector of feature values, ${y}_{i}$ is the class label that corresponds to ${x}_{i}$, $K$ is a parameter that specifies the desired number of weak learners, and ${f}_{k}$ is the prediction score of the *k*^*th*^ learner. Regularization is used to improve prediction performance as follows:

2 $$L(\Phi )= \sum_{i}l \left({\widehat{y}}_{i}, {y}_{i}\right)+\Omega ({f}_{k})$$

The part $\sum_{i}l \left({\widehat{y}}_{i}, {y}_{i}\right)$ is a loss function, which computes the difference between true (${y}_{i}$) and predicted (${\widehat{y}}_{i}$) class labels. The term $\Omega ({f}_{k})$ is a regularization function that penalizes the complexity of the model to prevent overfitting and can be formulated as follows:

3 $$\Omega \left({f}_{k}\right)=\gamma T+ \frac{1}{2} \lambda \sum_{j=1}^{T}{w}_{j}^{2}$$

where *T* is the number of leaf nodes in the weak learner and the parameters $\gamma$ and $\lambda$ control the regularization. $\gamma$ is the coefficient of the number of leaves and the $\lambda$ is the coefficient of the *l*-2 norm of the weights of all the leaf nodes.

To further control overfitting and reduce computational complexity, XGB utilizes several randomization approaches such as column subsampling and random subsampling. One of the challenges of using XGB is its relatively large number of model parameters. These parameters include the learningRate, nEstimators, maxDepth, minChildWeight, $\gamma$, subSample, and colSampleByTree. Optimizing these parameters is critical to achieving competitive prediction performance (Chen et al. 2020). To further improve prediction performance, this study attempts to fine-tune the parameters of XGB using ATSA.

### Parameter optimization

Different machine learning methods have different hyperparameters and some parameters can have infinite possible values. For instance, a Support Vector Machine model with a radial basis kernel has two real-value hyperparameters: the regularization parameter $C$ and the shape parameter $\gamma$ [43]. To overcome this challenge, we developed Adaptive Tabu Simulated Annealing (ATSA), a heuristic optimization algorithm designed to tune the parameters of machine learning models.

ATSA incorporates a strategy that blends the strengths of two well-established optimization methods, namely Simulated Annealing (SA) and Tabu Search (TS), to efficiently improve a starting solution to a given optimization problem. More on the theoretical development of SA and TS can be found in Kirkpatrick et al. [44] and [45] respectively. It starts with a randomly chosen solution and improves upon it by making small, random changes. The term “solution” in our application refers to a set of selected values for the parameters of the machine learning model being optimized. ATSA includes three strategies. The first is to accept worse solutions with a certain probability to avoid getting stuck in local optimal solutions. The second is to use an adaptive cooling schedule that adjusts the current search temperature based on the search trajectory. The adjustment can result with either cooling or possibly reheating. This improves the algorithm’s ability to explore better solutions. The third strategy is that ATSA keeps a list of recently tried solutions, called a tabu list, to prevent the algorithm from repeating itself [46]. Over time, the algorithm narrows its focus, honing in on the best solution found. This approach was used by Azizi and Zolfaghari [46] to solve job shop scheduling problems. In this study, we adapt ATSA and show that it is particularly useful for fine-tuning machine learning models by finding the best set of parameters that enhance machine learning model's performance.

**Fig. 3:**
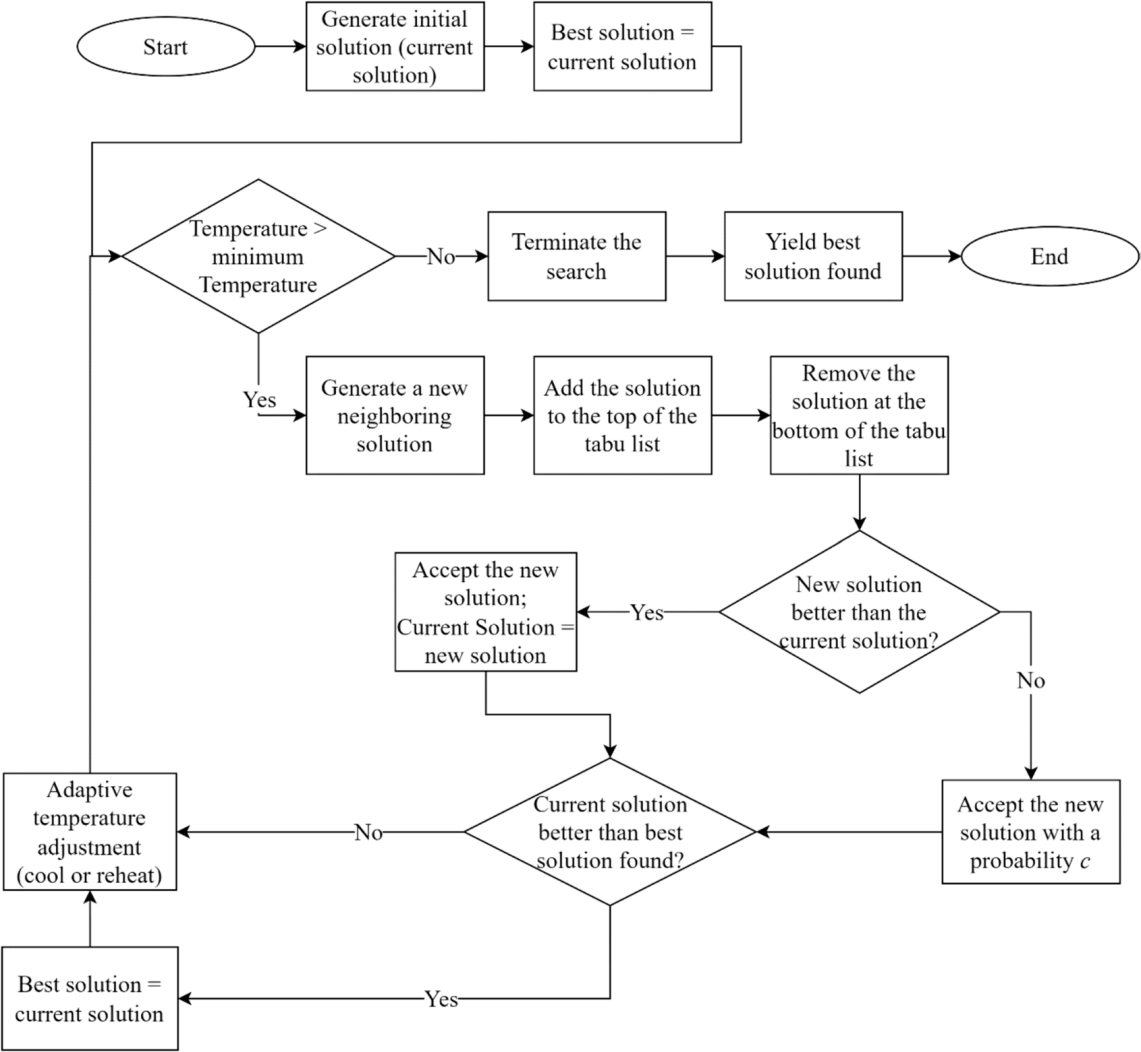
ATSA algorithm

As shown in Fig. 3, The algorithm search is initiated by guessing a starting point as the current best solution. It then checks whether the 'temperature'—a metaphor for willingness to explore—is still high. If it is, the algorithm suggests a new, nearby solution and notes it down in a list to remember it's been tried. It keeps track of these attempts in the tabu list, removing the oldest ones to make room for new ones. It evaluates the new solution; if it's better than the current one, it becomes the new current solution. If it's not better, it might still be considered, depending on a certain probability calculated based on the current search temperature. The next step is to determine whether to cool or reheat the search temperature using adaptive temperature control. This process repeats, with the algorithm becoming more selective over time, until the preset minimum search temperature is reached.

### Explanatory machine learning

Machine learning methods often work as a “closed-box” such that they produce models that are not interpretable. Recently, multiple approaches have been proposed to improve the interpretability of machine learning models. Example approaches are LIME [47], DeepLIFT [48], and Layer-Wise Relevance Propagation [49]. In these approaches, cooperative game theory equations are used to estimate the classic Shapley regression values [50]. Suppose that a feature value of an instance is considered as a “player”, while the prediction of the instance is considered as the “payout” in a game. The fair payout distribution among features is determined through Shapely values [51].

Another approach used for “closed-box” model interpretation is called *SHaply Additive exPlanations* (SHAP), which was proposed by [52]. In SHAP, the classic Shapley values approach is combined with other agnostic techniques such as LIME and DeepLIFT. Each feature is assigned an importance score, which represents the change in the expected model prediction, given the value of that feature. In SHAP, the importance score for instance $i$ of a feature $X$ (i.e., $X(i)$) is calculated based on its effect on model prediction. First, all features excluding $X$ are considered and then the deviation from the expected prediction is calculated when adding the instance $X(i)$ to all the features [52]. Thus, each score comes from an aggregated set of marginal contributions for each instance, which leads to the prediction of that instance. In this study, each patient’s admission disposition (i.e., SAT or LAMA) can be interpreted by aggregating the marginal contributions of features and calculating the importance of each feature by averaging the marginal contribution for all instances. In this paper, SHAP is used to interpret the developed model and determine the factors that affect the disposition status.

## Experimental results

This section presents the experimental results of the feature selection and prediction stages of the proposed framework. The importance and interpretation of the features of the best model are explained in this section as well.

### Experimental settings of model optimization

Eight XGB parameters are optimized in this study (See Table 4). The initial values of XGB parameters were obtained using uniform distributions. The parameter values are then updated throughout the optimization stage using the normal distribution. The mean and standard deviation of the normal distribution used are 0 and 1, respectively. The default values are used for the other parameters. The parameters are optimized using ATSA, and the results are compared. Table 5 shows the parameters of ATSA. We set ${T}_{min}$ to 2 for minimum temperature, enhancing solution space exploration; temperature increase rate *(β)* to 2, aiding in escaping local minima; Tabu list length of 20 to prevent redundant searches; and 300 algorithm iterations each allowing eight moves for comprehensive solution examination. Initial solutions were uniformly distributed for an unbiased start, while neighborhood searches used a normal distribution to focus on nearby solutions.

**Table 4:** Parameter ranges for XGB algorithms

Parameter	Possible range	Experimental range	Type
Number of estimators	[1, ∞)	[1, 50]	Integer
Maximum depth	[1, ∞)	[1, 50]	Integer
Maximum delta step	[1, ∞)	[1, 50]	Integer
Number of parallel trees	[1, ∞)	[1, 50]	Integer
Learning rate	(0,1]	(0,1]	Float
L1 regularization	(0,1]	(0,1]	Float
L2 regularization	(0,1]	(0,1]	Float
Gamma	(0, ∞]	(0,50]	Float

**Table 5:** SA and ASA parameters

Parameter	Value
${T}_{min}$	2
Rate of temperature increase *(β)*	2
Tabu length	20
Number of iterations	300
Number of moves	8
Initial solution generation	Uniform distribution
Neighborhood search	Normal distribution

### Missing data validation

Table 6 presents the MAPE that resulted from imputing different features using the KNN algorithm. The results showcase desirable accuracy in the imputed data, with MAPE values such as 2.04% for BMI, 0.81% for Systolic Blood Pressure, and a remarkably low 0.07% for Temperature in Fahrenheit. These examples illustrate the effectiveness of KNN imputation in our dataset, indicating that it is a reliable method for managing missing values in healthcare data.

**Table 6:** MAPE for KNN imputation

Feature	MAPE (%)
BMI	2.04
Systolic Blood Pressure	0.81
Diastolic Blood Pressure	0.98
Pulse Rate	1.52
Temperature in Fahrenheit	0.07
O2 Saturation	0.16
Respiratory Rate	0.95

### Feature selection results

Table 7 shows feature selection results. The selected features per each feature selection method are marked by (√). The last column of Table 7 shows the number of times each feature was selected by each of the four feature selection methods. Table 7 provides information about the importance of the selected features. Generally, the more often a feature is selected, the more important the feature is. For example, the Month of the Year is not selected by any method, which implies that this feature is not important with respect to the LAMA outcome. The last row in Table 7 shows the number of features selected by each feature selection method. For example, 12 features were selected by the DT_SFS method.

**Table 7:** Feature Selection results

Feature	DT_SFS	DT_SBS	RF_SFS	RF_SBS	Total
Age	√	√	√	√	4
Zip Code		√		√	2
BMI	√	√		√	2
Systolic Blood Pressure		√			1
Diastolic Blood Pressure		√	√	√	3
Pulse Rate		√			1
Temperature in Fahrenheit		√		√	2
O2 Saturation	√		√	√	3
Respiratory Rate	√	√	√	√	4
ESI Score	√	√	√	√	4
Day of the Month				√	1
Month of the Year					0
Hour of the Day	√				1
Waiting Time		√	√		2
ED Department Location ID10025	√		√		2
ED Department Location ID 10026	√		√		2
ED Department Location ID 15001	√	√	√	√	4
Patient Sex Male	√	√		√	3
Patient Ethnicity Not Hispanic or Latino			√		1
Patient Smoking Status Former Smoker	√		√		2
Patient Smoking Status Never Smoker	√		√	√	3
Total	12	12	12	12	

### Optimization results

**Fig. 4:**
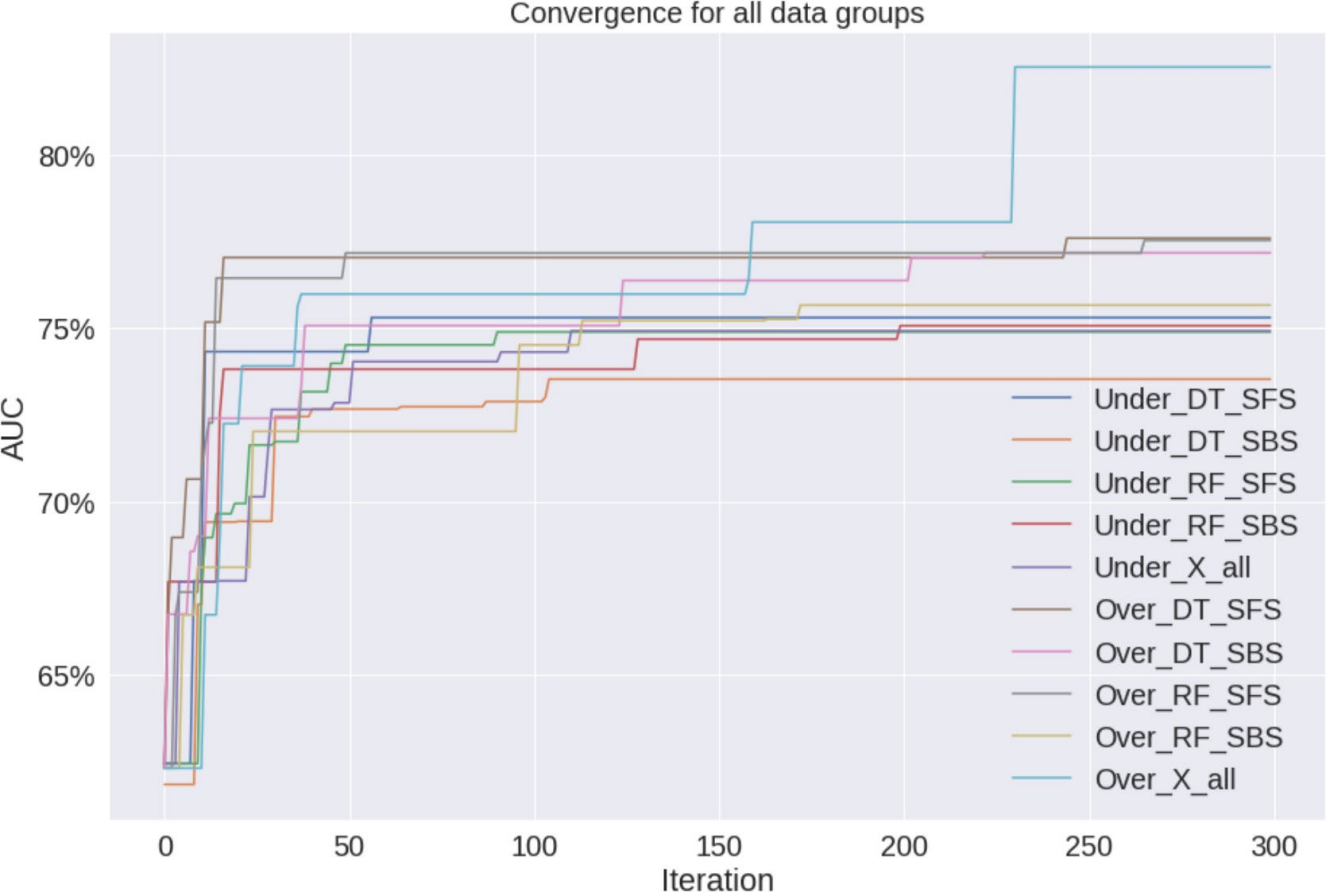
ATSA-XGB convergence of all data groups

ATSA is used to optimize the parameters of XGB. In addition to all the features (X_all), the data groups obtained from the feature selection stage are used to train and test the proposed algorithms under both oversampling and undersampling scenarios, yielding a total of 10 models. AUC is used as the main performance measure during the optimization stage. Figure 4 shows the optimization convergence of ATSA-XGB. Most of the models converged after 200 iterations. The AUC of the converged models ranged between 74 and 83%. Table 8 presents the optimized parameters for ATSA-XGB under the oversampling scenario. While most scenarios required a single estimator, DT_SFS required two. Maximum tree depth varied, with deeper trees needed for RF_SFS and X_all. Maximum delta step was typically 1, except for RF_SFS which was 2. The number of parallel trees was one, with the exception of DT_SFS and RF_SBS. Learning rates had wide ranges, highlighting the impact of feature selection on model tuning. L1 and L2 regularization values varied, indicating different needs for model simplicity and overfitting avoidance. Lastly, gamma values were generally high, suggesting a preference for fewer splits. These results emphasize the need for tailored XGBoost parameter tuning based on the specific feature selection method and dataset.

**Table 8:** Optimized parameters for ATSA-XGB

Parameter	DT_SFS	DT_SBS	RF_SFS	RF_SBS	X_all
Number of estimators	2	1	1	1	1
Maximum depth	1	1	2	1	4
Maximum delta step	1	1	2	1	1
Number of parallel trees	2	1	1	2	1
Learning rate	0.69	1.51	1.66	0.08	0.45
L1 regularization	1.66	2.00	1.06	0.38	1.15
L2 regularization	1.80	1.62	1.14	0.73	1.99
Gamma	1	1.16	8.79	9.34	1

### Prediction results

Table 9 presents the performance metrics of the proposed models during the training and testing phases with undersampling and oversampling conditions across various subsets of data derived from feature selection processes and the complete feature set (X_all). The performance of these models was assessed based on key metrics including Accuracy, AUC, Sensitivity, and Specificity, in the cross-validation and testing phases. We used 4-fold cross validation to develop the models. The mean AUC of training is utilized to decide the best model and optimize the models. Our analysis revealed that the model that is trained with all features (X_all), combined with oversampling, resulted as the best model. It recorded the highest mean AUC of 0.83 in the training phase and maintained a strong AUC of 0.76 during testing. This consistency in AUC performance across both phases reflects the model’s robustness and its capability of generalizing well to unseen data. While some models showed signs of overfitting, as indicated by a decline in performance from training to testing, the best model with oversampling displayed more resilience. The consistent performance of the best model in the testing phase exhibits strong generalization capabilities. This robustness, in conjunction with its superior AUC scores, clearly establishes it as the best model for our data.

**Table 9:** Models’ performance metrics during the training and testing phases

		Cross-validation				Testing			
	Model	Accuracy	AUC	Sensitivity	Specificity	Accuracy	AUC	Sensitivity	Specificity
Udnersampling	DT_SFS	0.69 ± 0.00	0.76 ± 0.01	0.83 ± 0.01	0.56 ± 0.00	0.66	0.74	0.69	0.66
	DT_sbs	0.68 ± 0.01	0.75 ± 0.01	0.81 ± 0.01	0.55 ± 0.02	0.56	0.75	0.82	0.55
	RF_SFS	0.68 ± 0.01	0.75 ± 0.02	0.76 ± 0.03	0.61 ± 0.03	0.62	0.74	0.73	0.62
	RF_sbs	0.68 ± 0.01	0.75 ± 0.01	0.82 ± 0.04	0.55 ± 0.04	0.50	0.74	0.87	0.50
	X_all	0.69 ± 0.02	0.76 ± 0.01	0.79 ± 0.02	0.59 ± 0.02	0.58	0.75	0.80	0.58
Oversampling	DT_SFS	0.70 ± 0.01	0.78 ± 0.00	0.71 ± 0.02	0.68 ± 0.01	0.68	0.74	0.66	0.68
	DT_sbs	0.70 ± 0.00	0.77 ± 0.00	0.76 ± 0.01	0.64 ± 0.00	0.65	0.75	0.72	0.65
	RF_SFS	0.71 ± 0.00	0.78 ± 0.00	0.86 ± 0.00	0.56 ± 0.00	0.56	0.75	0.83	0.55
	RF_sbs	0.69 ± 0.00	0.76 ± 0.00	0.79 ± 0.04	0.60 ± 0.04	0.64	0.74	0.73	0.64
	X_all	0.75 ± 0.00	0.83 ± 0.00	0.84 ± 0.01	0.67 ± 0.01	0.56	0.76	0.82	0.56

### Model interpretation

**Fig. 5:**
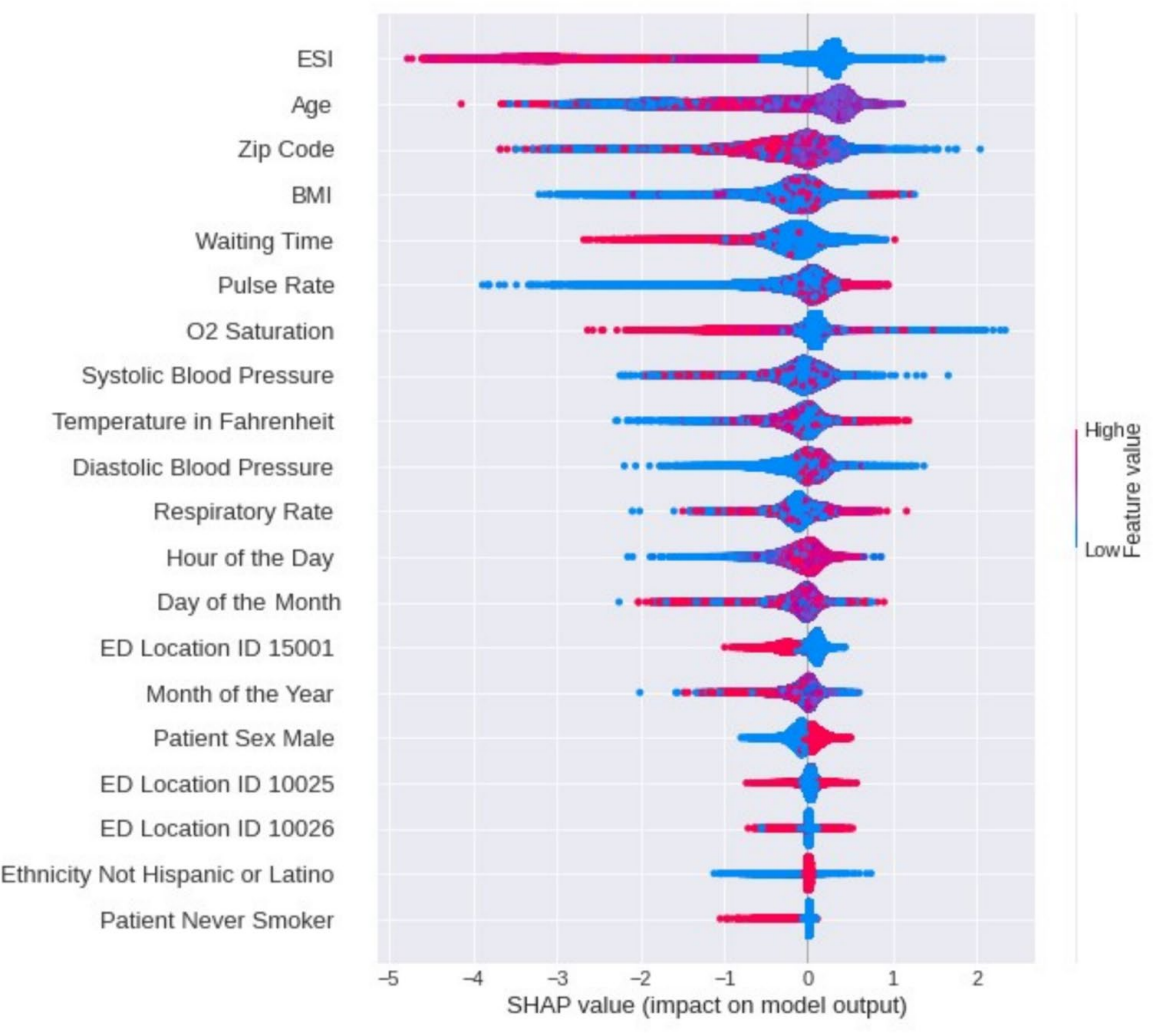
SHAP feature importance results

To interpret the data features used in this study, we consider both the model that includes all the features and the best model found using selected features and hyperparameter optimization. The best model has a testing AUC 76% and was trained and tested using ASA-XGB on all the features (i.e., X_all) based on the oversampling scenario (see Table 9). The model with optimized parameters is used along with the SHAP algorithm. The Python SHAP library is used to obtain feature scores and perform model interpretation. Figure 5 shows the SHAP graph for the best model. The SHAP summary plot offers an insightful visualization of feature importance and their directional impact on a prediction model. On the y-axis, features are ranked by their influence on the model, while the x-axis includes SHAP values, indicating the extent to which each feature shifts the model’s output from a base value. Individual data points, colored to represent feature values (red for high, blue for low), scatter along the horizontal axis, revealing the impact magnitude and direction: rightward for positive and leftward for negative contributions. The density of these points reflects the consistency of a feature’s effect across the dataset instances. Such a plot not only identifies key predictive features but also encapsulates the complex interplay between them, enabling a granular understanding of the model's behavior.

By inspecting the SHAP summary plot in Fig. 5, we notice that the ESI score is the most influential variable, where higher scores (i.e., low acuity patients) are associated with patients that are more likely to leave against medical advice (class 1). The age feature showed a diverse range of effects on the predictions, with no immediate trend discernible, suggesting a complex relationship between age and patient disposition that warrants further investigation.

Regarding the influence of BMI on patient outcomes, the SHAP summary plot indicates a nuanced impact. The distribution of SHAP values for BMI stretched across both sides of the zero line, implying that the relationship between BMI and patient decisions upon ED discharge is not linear or straightforward. While the plot does not immediately reveal a distinct pattern, it is evident that both higher and lower BMI values can significantly sway the model's predictions, suggesting that both underweight and overweight patients may have different but notable reasons for their disposition from the ED.

For vital signs, the SHAP analysis indicates that high pulse and respiratory rates are associated with an increased likelihood of patients LAMA. These elevated vital signs could indicate a state of medical distress or anxiety, which may influence a patient's decision to leave the emergency department prematurely. The concentration of higher SHAP values for these vital signs on the right side of the plot underscores their significance in the predictive model, suggesting that when these measurements are high, they are strong predictors for a LAMA outcome. Both systolic and diastolic blood pressure measurements have a complex relationship with the likelihood of a patient LAMA. While the plot does not explicitly indicate a clear directional trend, the spread of SHAP values for these vital signs across the zero line implies that both high and low blood pressure readings can influence the model's prediction. It can be inferred that extreme values in either direction may contribute to a patient's decision for LAMA. This happens potentially due to the symptoms or risks associated with uncontrolled hypertension or hypotension, which can impact their perceived need for immediate care or discomfort with staying in the emergency department. The SHAP summary plot shows that lower values of oxygen saturation are associated with a higher likelihood of patients LAMA. This trend suggests that patients with lower oxygen saturation levels, which could be indicative of respiratory distress or other serious health conditions, may be more prone to opt for LAMA. This counterintuitive outcome might reflect underlying challenges in the healthcare setting, such as patient discomfort or dissatisfaction with care, particularly in those presenting with critical signs that necessitate immediate and effective treatment.

**Fig. 6:**
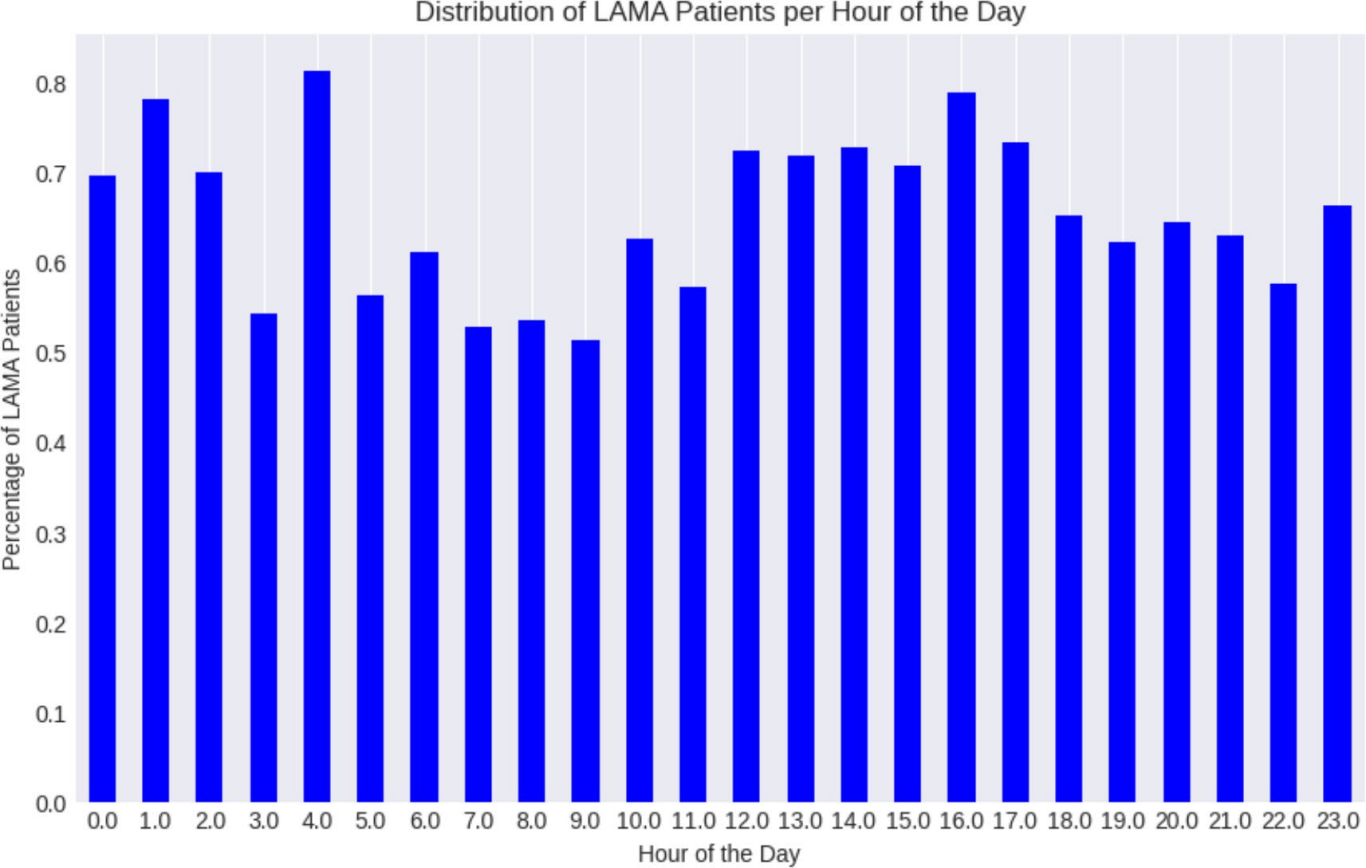
Distribution of LAMA throughout hours of the day

For the hour of the day, day of the month, and month of the year features, the SHAP highlights that the effect of the hour of the day on LAMA is not linear. Late afternoon hours (middle of SHAP bar) are associated with a higher risk of LAMA, whereas early morning hours or late evening show a lower risk. This is consistent with the distribution of LAMA during the day (See Fig. 6) Conversely, lower values for the month of the year, likely corresponding to winter months, also show a higher tendency for LAMA. This could be due to seasonal factors like increased incidence of illnesses, weather-related challenges, or holiday-related stressors impacting patient decisions. These findings underscore the significant influence of temporal factors like time of day and seasonality on patient behavior in emergency department settings. The day of the month does not exhibit a clear pattern.

For the waiting time feature, the SHAP values predominantly show negative values, indicating that longer ED front-end waiting times are associated with a decreased risk of LAMA. However, it is important to note the wait time feature in our model does not capture the entire waiting experience in the ED such as back-end waiting times. The absence of back-end waiting time data in our model is a limitation that should be considered when interpreting these results.

For the ED location IDs, the SHAP graph shows differentiated impacts on the likelihood of patients LAMA. The plot illustrates that certain ED locations are more strongly associated with LAMA. For instance, ED Location ID 10025 shows a cluster of higher SHAP values, suggesting that this location has a higher influence on the prediction of LAMA outcomes. This could be due to various location-specific factors such as patient population characteristics, resource availability, or operational practices that influence a patient's experience and decision to leave. Meanwhile, other location IDs, such as 10026, appear to have less influence, as indicated by the SHAP values closer to zero. This variation underlines the importance of considering location-specific dynamics when addressing the factors that contribute to LAMA incidents.

For the demographic features, the feature Patient Sex Male showed a slight tendency towards influencing patients to LAMA. This suggests that male patients may have a marginally higher risk of LAMA. Conversely, the feature 'Ethnicity Not Hispanic or Latino' predominantly populated the negative side of the SHAP value spectrum, implying a decreased likelihood of LAMA for this demographic group, which could reflect underlying socio-cultural dynamics in healthcare utilization. Similarly, the status of 'Patient Never Smoker' was associated with a tendency to be seen and treated, as evidenced by SHAP values leaning negatively. These insights emphasize the importance of demographic and behavioral factors in patient decision-making processes within emergency care contexts. The zip code feature shows a mixed influence on the model's predictions. The diverse SHAP values indicate that certain zip codes correlate with a higher likelihood of patients leaving against medical advice, while others correlate with patients being seen and treated. This suggests that the zip code is a proxy for more complex socio-economic and environmental factors that can influence patient outcomes in the context of health care delivery. This can be investigated as a future direction of this study.

**Fig. 7:**
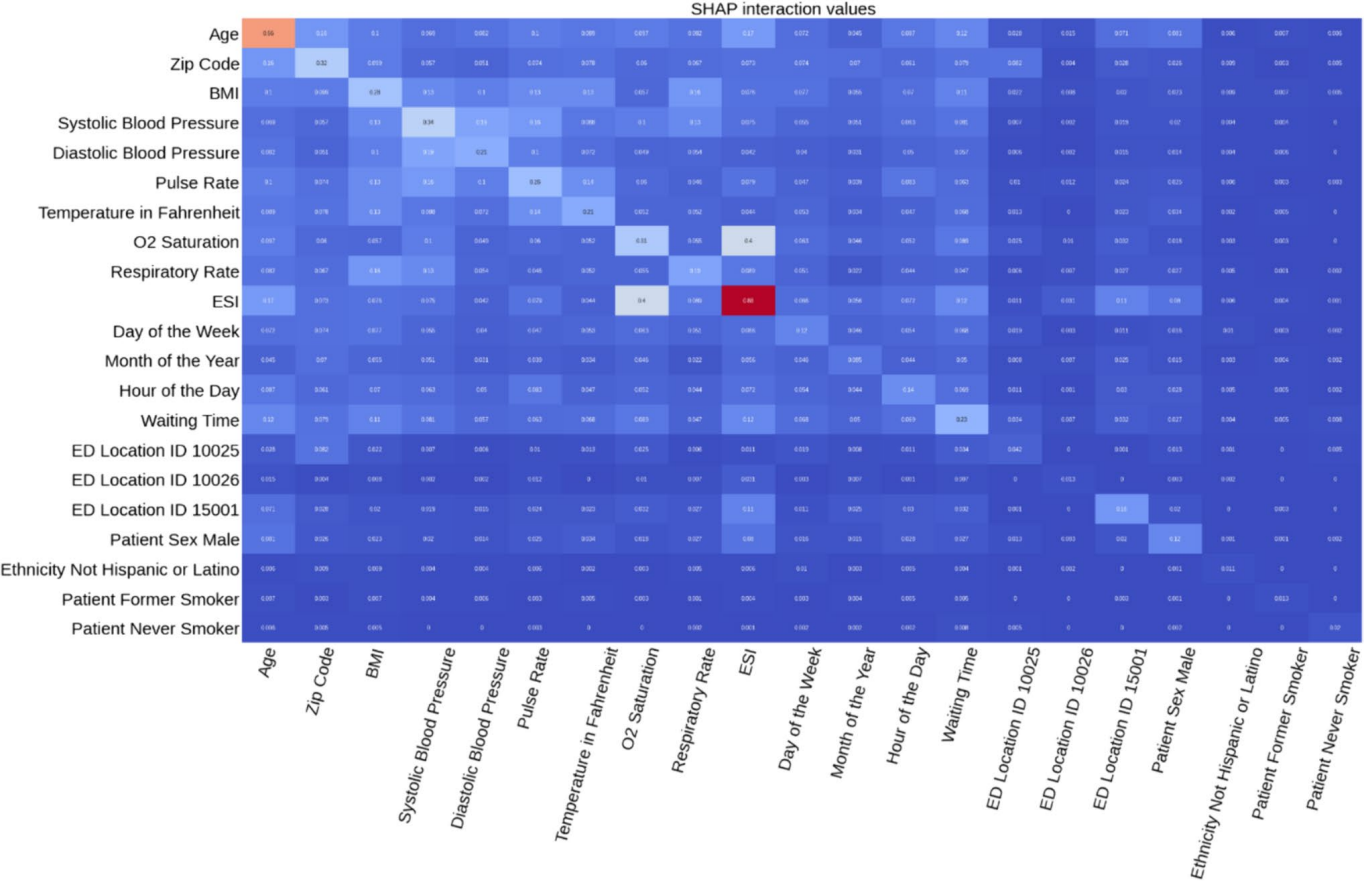
SHAP main and interaction effect

Figure 7 shows a heatmap plot of the SHAP main and interaction effects. The plot shows that most of the feature pairs exhibit moderate interaction effects, as indicated by the predominance of lighter-colored cells, whereas numerous interactions appear negligible, suggested by darker hues. Many cells have lighter colors, indicating that while there are interactions present between the features, most are moderate rather than strong. This suggests that for many feature pairs, the interaction does not drastically change the model's output compared to their individual effects. Some cells are dark blue or close to zero, suggesting that certain pairs of features have little to no interaction effect on the model's output.

Comparing our study with existing studies in the literature, our study retains several significant features identified in existing literature, such as demographic information and vital signs [20, 21], while introducing additional relevant features such as ESI scores and specific location IDs. Insurance status was a common feature discussed in the literature [22] but not considered in our model due to the limited data we obtained from our partner hospital.

### Managerial impact

This study provides a prediction tool to anticipate the LAMA patients at the time of triage. This early detection allows for immediate intervention strategies, such as expediting care for those at higher risk or providing additional support and communication to address concerns that might lead to LAMA. The integration of this model into the triage process has potential implications for enhancing the overall ED efficiency and patient satisfaction. By proactively managing potential LAMA cases, EDs can improve throughput, reduce overcrowding, and enhance the quality of care, leading to better patient outcomes and experiences. From a financial perspective, reducing LAMA rates can decrease the incidence of unbilled services and improve the hospital's revenue cycle.

Furthermore, this study leverages explainable AI tool, named SHAP, to interpret the features that affect LAMA patients. This provides managers with actionable insights into the factors influencing LAMA incidents. For example, the proposed model indicated that factors such as ESI score, patient, vital signs, and waiting times play critical roles in predicting LAMA likelihood. For instance, longer waiting times are associated with increased LAMA risks, suggesting that managerial efforts to reduce wait times could directly impact patient retention. Similarly, the time of day influences LAMA occurrences, guiding managers to allocate resources more efficiently during high-risk periods. Zip codes, reflecting the patient’s socio-economic status, also correlates with LAMA rates, indicating a potential need for targeted community initiatives or resource allocation strategies. Gender and smoking status provide further granularity, revealing subtle trends that can refine patient communication and support strategies. Notably, the predictive model highlights the importance of ESI scores, with higher scores correlating with a greater likelihood of LAMA. This insight can prompt ED managers to prioritize high-risk patients for expedited care. In essence, the predictive model serves as a strategic tool for healthcare administrators, enabling the optimization of operational efficiency, patient throughput, and resource allocation. By preemptively identifying patients at risk of LAMA, the model facilitates improved patient care management, potentially enhancing patient outcomes and satisfaction while concurrently safeguarding the hospital's financial interests by minimizing incomplete treatments. In summary, the predictive model offers a strategic, data-driven approach to anticipate and manage LAMA cases in EDs, aligning operational efficiency with optimal patient care, and addressing both clinical and administrative challenges in emergency medicine.

## Conclusions and future work

This paper presents an explanatory machine learning framework that can be used as a decision-support tool to help healthcare practitioners and administrators early identify LAMA patients in EDs. Early identification of LAMA patients allows for taking appropriate measures to prevent patients from leaving the ED before receiving necessary care. This can improve the quality and efficiency of ED care and prevent adverse patient outcomes such as readmission and mortality. The proposed framework provides theoretical and practical contributions to the area of healthcare analytics. The theoretical contribution comes from proposing the use of ATSA for optimizing XGB parameters. Although the proposed algorithm is used for optimizing XGB parameters, it can be used to optimize the parameters of any machine learning algorithm.

The practical contribution of this work is the application of machine learning to explain the effect of a patient's demographic information and vital signs on the rate of LAMA, which is an important quality and efficiency measure of ED processes. We developed various models based on different data groups that resulted from different feature selection procedures. The best model included all the features and resulted in an accuracy, AUC, sensitivity, and specificity of 56%,76%, 82%, and 56%, respectively. For future work, natural language processing can be used to analyze clinical notes, which may help in better understanding the causes of why patients leave the ED before treatment is complete. Another future extension of this research is to develop a LAMA risk scoring model, which would provide insights into the level of LAMA risk posed by individual patients. In the future, we will also consider other factors that are related to LAMA, including social determinant of heath.

## Data Availability

Data will be shared on request to the corresponding author with permission of the partner hospital.
